# The role of *Akkermansia muciniphila* in cancer: Mechanisms, therapeutic potential, and challenges

**DOI:** 10.1002/imo2.70010

**Published:** 2025-03-27

**Authors:** Xi Chen, Yao Li, Guoli Wei, Zihan Zheng, Wei Liu, Mengyuan Li, Xuening Dai, Boyuan Liu, Rongling Zhong, Juan Ye

**Affiliations:** ^1^ Affiliated Hospital of Integrated Traditional Chinese and Western Medicine Nanjing University of Chinese Medicine Nanjing Jiangsu China; ^2^ Department of Pharmacology, School of Pharmacy Nanjing University of Chinese Medicine Nanjing Jiangsu China; ^3^ Department of Oncology, Affiliated Hospital of Integrated Traditional Chinese and Western Medicine Nanjing University of Chinese Medicine Nanjing Jiangsu China; ^4^ Department of Oncology Nanjing Lishui District Hospital of Traditional Chinese Medicine Nanjing Jiangsu China; ^5^ Department of Animal & Dairy Science University of Wisconsin‐Madison Madison Wisconsin USA; ^6^ Department of Ophthalmology The Affiliated Suqian First People's Hospital of Nanjing Medical University Suqian Jiangsu China; ^7^ Core Laboratory, Sir Run Run Hospital Nanjing Medical University Nanjing Jiangsu China

## Abstract

*Akkermansia muciniphila* (*A. muciniphila*), regarded as a promising candidate for next‐generation probiotic applications, predominantly inhabits the intestinal mucus layer, where it plays a crucial role in maintaining gut barrier integrity and modulating immune responses. Recently, the bacterium has been recognized for its ambivalent influence on cancer, impacting both tumor progression and therapeutic interventions. Research indicates that *A. muciniphila* might possess both tumorigenic and anticancer capabilities, influenced by factors such as the composition of the gut microbiota, dietary modifications, and immune modulation. There is a compelling need for further studies to uncover the precise mechanisms and optimal use of *A. muciniphila* in oncology and beyond.

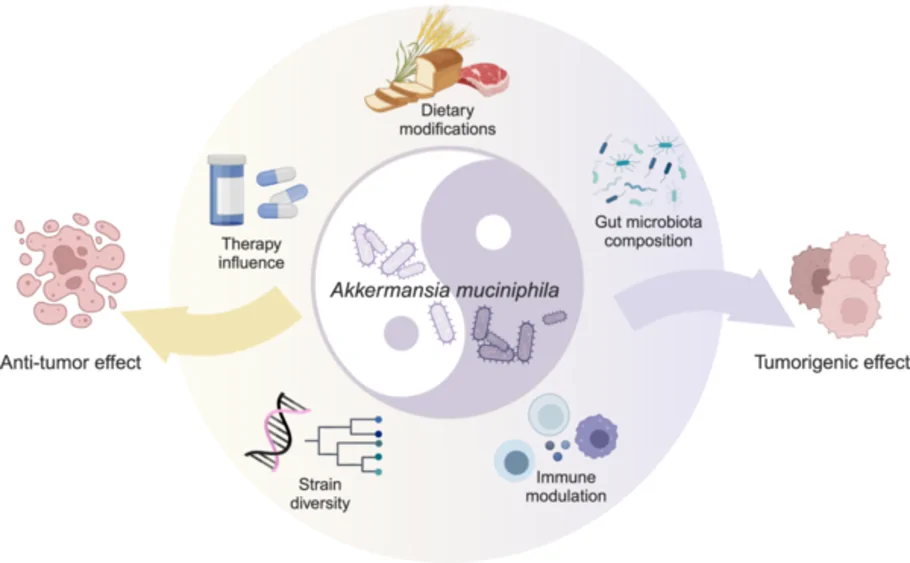

The human microbiome has emerged as a crucial determinant of health, with the gut microbiota playing a significant role in various diseases, including cancer [1]. Among the many microbiota inhabitants, *Akkermansia muciniphila* (*A. muciniphila*), a Gram‐negative, mucin‐degrading bacterium, has attracted considerable attention due to its multifaceted roles in gut health and its ambivalent relationship with cancer. This Commentary delves into the dual nature of *A. muciniphila*'s influence on cancer, exploring its mechanisms in both promoting and inhibiting tumorigenesis, as well as its therapeutic potential and the challenges that remain for clinical application.

## 1 *AKKERMANSIA MUCINIPHILA*: PHYSIOLOGICAL ROLES IN CANCER THERAPY

**FIGURE 1:**
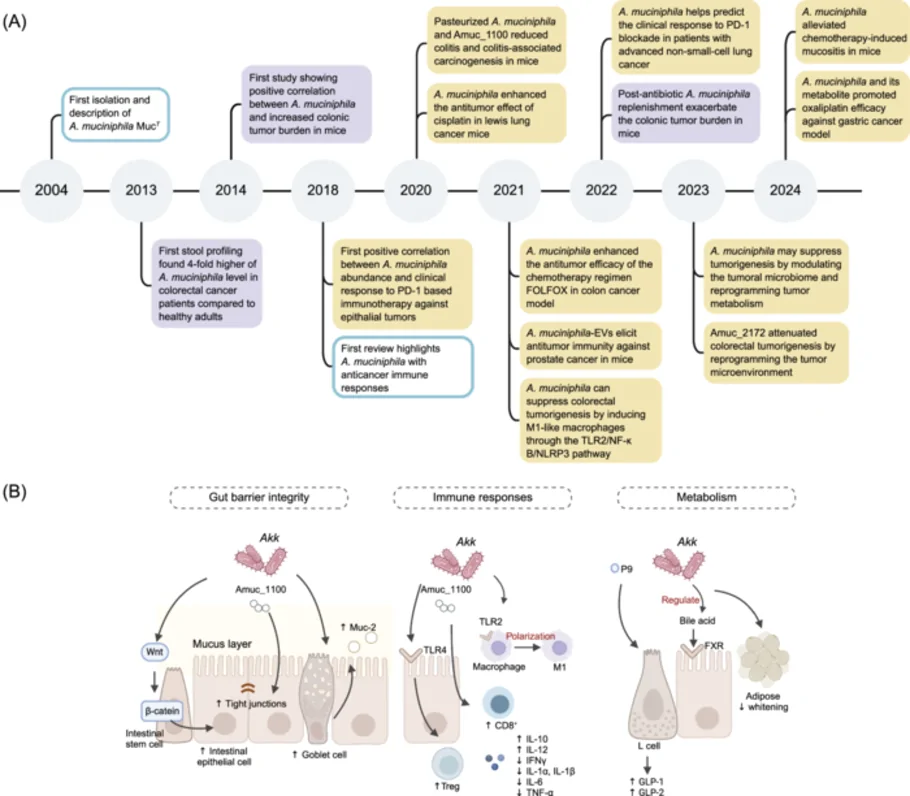
*Akkermansia muciniphila*'s role in cancer research and physiological functions. (A) Key milestones in the research progress of *A. muciniphila* in cancer studies. (B) Physiological functions of *A. muciniphila*.

In recent years, as research has progressed, Figure 1A illustrates the key milestones in the research progress of *A. muciniphila* in cancer studies, and its potential mechanisms of action have gradually been uncovered. *A. muciniphila* is a Gram‐negative bacterium that resides in the mucosal layer of the human intestine, where it degrades mucin, a key component of the intestinal mucus. This degradation not only helps maintain gut integrity but also promotes a healthy microbiome, which is crucial in preventing inflammatory diseases and cancer [2]. By enhancing the mucus layer and supporting tight junction formation between epithelial cells, *A. muciniphila* acts as a barrier against pathogens and harmful stimuli that could contribute to cancer development [3].

Moreover, *A. muciniphila* plays a key role in modulating the immune system [4]. It has been shown to activate regulatory T cells (Tregs), which are critical for maintaining immune tolerance and preventing chronic inflammation—a key driver of cancer progression. Additionally, *A. muciniphila* has been linked to enhanced immune checkpoint inhibition efficacy, particularly in cancers treated with immune checkpoint inhibitors like anti‐PD‐1/PD‐L1 antibodies. This bacterium influences the gut immune system, potentially improving the host's response to cancer treatments, and making it an exciting candidate for combination therapies with immune checkpoint inhibitors. The multifaceted physiological roles of *A. muciniphila* are further illustrated in Figure 1B.

## 2 THE DUAL ROLE OF *AKKERMANSIA MUCINIPHILA* IN CANCER PROGRESSION

**FIGURE 2:**
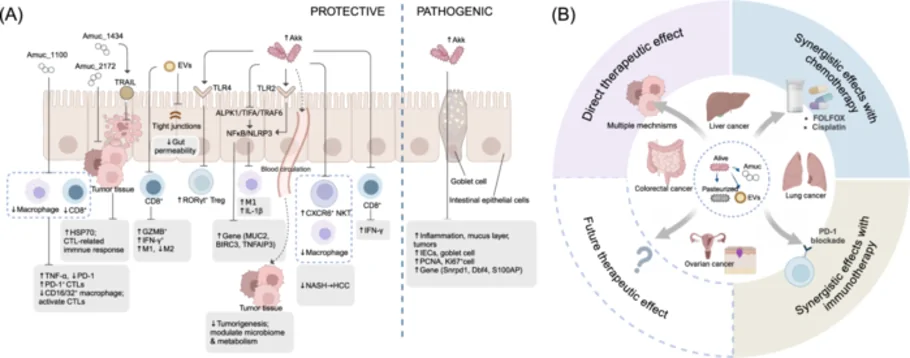
Overview of the relationship between *Akkermansia muciniphila* and cancer. (A) Major mechanisms associated with the dual effects of *A. muciniphila* or related substances in tumor. By regulating RORγt^+^ regulatory T‐cell responses through binding to TLR4, *A. muciniphila* reduces susceptibility to colon inflammation. *A. muciniphila* activates native macrophage into M1‐like TAMs that release IL1B to inhibit colonic tumorigenesis in mice. TLR2 acts as the macrophage pattern recognition receptor to *A. muciniphila*, activates the NF‐ κB/NLRP3 pathway, and stimulates the acquisition of the M1 macrophage phenotype. Also, *A. muciniphila* could promote intestinal homeostasis through activating the ALPK1/TIFA/TRAF6 axis. Through reducing macrophage infiltration and enhancing hepatic natural killer T (NKT) cell populations, *A. muciniphila* treatment mitigated nonalcoholic steatohepatitis (NASH) progression to hepatocellular carcinoma (HCC). *A. muciniphila* suppressed ovarian cancer progression in mice, with enhanced IFN‐γ secretion of CD8^+^ T cells and their tumor‐killing properties. Beyond the direct interaction with immune pathways, the capacity of *A. muciniphila* to influence tumorigenesis may also be mediated through migrating into blood circulation and colonizing tumor tissues. Supplementation with Amuc_1100 blunted tumourigenesis by expanding cytotoxic T lymphocytes (CTLs) in the colon and mesenteric lymph nodes (MLN), and activated CTLs in MLN further induce TNF‐α and downregulate PD‐1. Amuc_2172 has been implicated in attenuating colorectal tumorigenesis by reprogramming the tumor microenvironment through inducing HSP70 secretion and promoting CTL‐related immune response. Amuc_1434 suppresses LS174T cell viability via upregulating the expression of tumor‐necrosis‐factor‐related apoptosis‐inducing ligand (TRAIL), thereby activating the death receptor pathway and mitochondrial pathway of apoptosis, which was related to its ability to degrade Muc2. Extracellular vesicles derived from *A. muciniphila* (EVs) can influence gut permeability by regulating tight junctions. EVs could elevate the proportion of granzyme B‐positive (GZMB^+^) and interferon γ‐positive (IFN‐γ^+^) lymphocytes in CD8^+^ T cells and cause macrophage recruitment, with increased tumor‐killing M1 macrophages and decreased immunosuppressive M2 macrophages. However, *A. mucinipila* may induce pathogenic effects in mice by boosting the initial inflammatory level and the proliferation of intestinal epithelial cells (IECs), increase in goblet cell density, and mucus layer thickness. PCNA expression, Ki67^+^ proliferating cells, and gene expression of Snrpd1, Dbf4, and S100A9 as proliferation‐associated molecules were higher in *A. muciniphila*‐received mice. (B) Direct effect and synergistic effects of *A. muciniphila* in tumor therapy.

Despite its potential benefits, *A. muciniphila* also exhibits a dual role in cancer progression, which complicates its application as a cancer therapeutic. As shown in Figure 2, while *A. muciniphila* has shown promise in immune modulation and cancer suppression; it can also promote tumorigenesis under specific conditions.

On one hand, studies indicate that *A. muciniphila* enhances antitumor immunity by stimulating the M1‐like macrophage polarization and promoting NLRP3 inflammasome activation [5]. These immune‐modulatory effects contribute to the bacterium's ability to suppress colorectal cancer (CRC) progression [6, 7]. Furthermore, animal models have shown that *A. muciniphila* supplementation leads to a reduction in tumor growth, enhanced T‐cell infiltration into tumors, and improved response to chemotherapy [8, 9]. This suggests that *A. muciniphila* might have therapeutic benefits, particularly when combined with immunotherapies, through its ability to activate immune responses and modulate the tumor microenvironment [10].

On the other hand, recent evidence suggests that *A. muciniphila* can exacerbate cancer progression under certain conditions. Excessive colonization of the gut by *A. muciniphila* has been associated with the disruption of the gut barrier in animal models, leading to increased systemic inflammation and promoting the development of CRC [11]. In patients with inflammatory bowel disease (IBD)—a known risk factor for CRC—elevated levels of *A. muciniphila* have been linked to increased susceptibility to tumorigenesis. This highlights the importance of maintaining a balanced microbiome, as *A. muciniphila*'s effects on cancer could be either beneficial or harmful depending on the overall gut microbial composition and immune status of the host.

## 3 IMMUNOTHERAPY AND *AKKERMANSIA MUCINIPHILA*: ENHANCING CANCER TREATMENT

One of the most promising aspects of *A. muciniphila* in cancer therapy is its potential to enhance the efficacy of immunotherapies [12]. Recent studies have shown that higher levels of *A. muciniphila* in the gut correlate with better responses to PD‐1 inhibitors in patients with non‐small‐cell lung cancer [13] and renal cell carcinoma. This bacterium is thought to influence the gut microbiota in a way that promotes immune cell activation, leading to improved antitumor immunity.

In a groundbreaking study, the combination of *A. muciniphila* with immune checkpoint inhibitors significantly increased progression‐free survival in patients, highlighting the potential of *A. muciniphila* as an adjunct to immunotherapy. Similarly, animal models have shown that *A. muciniphila* supplementation leads to enhanced immune checkpoint inhibition efficacy, particularly in CRC, where it modulates the immune response and improves tumor control. These findings suggest that combining *A. muciniphila* with immunotherapies could represent a novel therapeutic strategy to boost the body's natural immune defenses against cancer.

Moreover, *A. muciniphila* has been shown to improve the efficacy of chemotherapy in various models [14, 15, 16]. The addition of *A. muciniphila* to chemotherapy regimens like cisplatin or the FOLFOX regimen (oxaliplatin, fluorouracil, calcium folinate) has resulted in better outcomes, including reduced tumor growth and increased immune cell infiltration into tumors [17]. *A. muciniphila*'s ability to enhance chemotherapy effectiveness while also protecting against radiation‐induced gut toxicity further strengthens its potential as a therapeutic agent in cancer treatment.

## 4 CHALLENGES IN THE CLINICAL APPLICATION OF *AKKERMANSIA MUCINIPHILA*

Despite the promising therapeutic effects of *A. muciniphila*, several challenges must be addressed before it can be clinically applied. One of the key hurdles is ensuring the survival of *A. muciniphila* during oral administration. The acidic conditions of the stomach and digestive enzymes pose significant barriers to the viability of this bacterium. Developing stable formulations that protect *A. muciniphila* and ensure its effective delivery to the intestines is essential for its clinical application. Furthermore, the strain‐specific effects of *A. muciniphila* complicate its use. Not all strains of *A. muciniphila* exhibit the same therapeutic properties, and understanding which strains are most effective in cancer therapy is crucial.

Another major challenge is the variability in individual responses to *A. muciniphila* supplementation. The composition of the gut microbiota differs significantly among individuals, influenced by factors such as diet, genetics, and environmental exposures. These variations can impact how *A. muciniphila* interacts with the host's immune system and cancer therapies, making it essential to personalize treatment strategies. Microbiome profiling and dietary interventions tailored to enhance *A. muciniphila* abundance in specific cancer patient populations could improve therapeutic outcomes [18].

Finally, while preclinical studies show promising results, further clinical trials are needed to evaluate the safety [19], optimal dosing, and long‐term effects of *A. muciniphila* in cancer patients. Understanding how *A. muciniphila* interacts with other cancer therapies and the gut microbiota will be crucial for its successful integration into clinical practice.

## 5 FUTURE DIRECTIONS

The future of *A. muciniphila* in cancer therapy is promising, but several key areas need to be addressed to fully realize its potential:

Strain‐specific mechanisms: More research is needed to identify which strains of *A. muciniphila* are most effective in cancer therapy. Investigating the genetic and metabolic profiles of different strains will help select the most beneficial strains for cancer treatment.

Personalized microbiome‐based therapies: Given the variability in patient responses to *A. muciniphila*, microbiome‐based interventions tailored to individual patients could enhance therapeutic outcomes. Personalized dietary strategies or probiotics aimed at optimizing *A. muciniphila* levels should be explored to maximize its cancer‐fighting potential.

Clinical integration and safety evaluation: Large‐scale clinical trials are essential to assess the safety, efficacy, and optimal delivery methods for *A. muciniphila* in cancer therapy. These trials will help establish protocols for its use in combination with other cancer treatments, such as immunotherapies and chemotherapy.

Combining *A. muciniphila* with chemotherapies and immunotherapies: Future studies should focus on the synergistic effects of *A. muciniphila* with existing cancer therapies, particularly immunotherapies and chemotherapy regimens. These studies could provide valuable insights into how to enhance treatment efficacy and minimize side effects.

## 6 CONCLUSION

*A. muciniphila* represents a promising next‐generation probiotic with significant potential in cancer prevention and therapy. Its dual role in cancer progression—both as a suppressor and a potential promoter—underscores the complexity of its effects. By addressing the challenges related to its clinical application, such as strain‐specific effects, delivery methods, and patient variability, *A. muciniphila* could 1 day become a key player in cancer treatment, enhancing the efficacy of existing therapies and improving patient outcomes. Future research is essential to uncover the full therapeutic potential of this remarkable bacterium and to develop strategies for its safe and effective clinical use.

## AUTHOR CONTRIBUTIONS

**Xi Chen**: Writing—original draft; writing—review and editing. **Yao Li**: Writing—review and editing; writing—original draft. **Guoli Wei**: Writing—review and editing; writing—original draft. **Zihan Zheng**: Writing—review and editing. **Wei Liu**: Writing—review and editing. **Mengyuan Li**: Writing—review and editing. **Xuening Dai**: Writing—review and editing. **Boyuan Liu**: Writing—review and editing. **Rongling Zhong**: Writing—review and editing. **Juan Ye**: Writing—original draft; conceptualization; writing—review and editing; funding acquisition.

## CONFLICT OF INTEREST STATEMENT

The authors declare no conflicts of interest.

## ETHICS STATEMENT

The authors have nothing to report.

## Data Availability

Data sharing is not applicable to this article as no datasets were generated or analyzed during the current study. No new data were created or analyzed in this study. Supplementary materials (graphical abstract, slides, videos, Chinese translated version and update materials) may be found in the online DOI or iMeta Science http://www.imeta.science/imetaomics/.
